# First Case of Subcutaneous Mycoses Caused by *Dirkmeia churashimaensis* and a Literature Review of Human *Ustilaginales* Infections

**DOI:** 10.3389/fcimb.2021.711768

**Published:** 2021-11-02

**Authors:** Fengming Hu, Chong Wang, Peng Wang, Lei Zhang, Qing Jiang, Abdullah M. S. Al-Hatmi, Oliver Blechert, Ping Zhan

**Affiliations:** ^1^ Department of Integrated Chinese and Western Medicine, Dermatology Hospital of Jiangxi Province and Jiangxi Dermatology Institute, Nanchang, China; ^2^ Dermatology Department, Liaocheng People’s Hospital, Liaocheng, China; ^3^ Dermatology Department, The Second People’s Hospital of Guiyang, Guiyang, China; ^4^ Natural & Medical Sciences Research Center, University of Nizwa, Nizwa, Oman; ^5^ Department of Biological Sciences & Chemistry, College of Arts and Sciences, University of Nizwa, Nizwa, Oman; ^6^ Centre of Expertise in Mycology Radboudumc/CWZ, Nijmegen, Netherlands; ^7^ The Institute of Clinical Medicine & Dermatology Department, Jiangxi Provincial People’s Hospital Affiliated to Nanchang University, Nanchang, China

**Keywords:** *Dirkmeia*, *Pseudozyma*, *Moesziomyces*, Ustilaginales, infection, subcutaneous

## Abstract

**Objective:**

*Dirkmeia churashimaensis*, belonging to Ustilaginales fungi, has never been reported as clinical pathogenic until very recently. In this study, we report an unusual subcutaneous infection with *Dirkmeia churashimaensis* and reviewed all human Ustilaginales infections. The aim is to better understand their epidemiology, infection type, risk factors, and the sensitivity to antifungal agents.

**Methods:**

An 80-year-old female farmer developed extensive plaques and nodules on her left arm within 2 years. Pathological and microbiological examinations identified a new pathological agent, *Dirkmeia churashimaensis*, as the cause of this infection. The patient was successfully cured by oral itraconazole. We reviewed a total of 31 cases of Ustilaginales cases, among of which only three were skin infections.

**Results:**

Local barrier damage (i.e., surgery, trauma, and basic dermatosis) and systemic immunodeficiency (i.e., preterm and low birthweight, Crohn’s disease, malignant cancer, and chemotherapy) are risk factors for Ustilaginales infection. The D1/D2 and ITS regions are the frequently used loci for identifying the pathogens together with phenotype. Most patients could survive due to antifungal treatment, whereas seven patients died. Amphotericin B, posaconazole, itraconazole, and voriconazole showed good activity against these reported strains, whereas fluconazole, 5-flucytosine, and echinocandins usually showed low susceptibility. Itraconazole had good efficiency for subcutaneous infections.

**Conclusions:**

The present case study and literature review reveal that Ustilaginales can be opportunistic pathogenic normally in immunocompromised and barrier damage people. A proper identification of fungi can be crucial for clinical treatment, and more data of antifungal are needed for choice of medication against this kind of infections.

## Introduction

Ustilaginales is a large order within the smut fungi (Ustilaginomycetes) including many species forming blackish to brownish powdery spore mass in different organs of plants (Kruse et al., 2017). Most of them are typically parasitic and some species are important pathogens of plants, such as corn smut (*Pseudozyma prolifica*) and wheat smut (*Ustilago nuda*) (Kruse et al., 2017) (Morita et al., 2011). Ustilaginales were occasionally isolated from clinical context, mainly consisting of species within *Pseudozyma* and *Moesziomyces* (Telles et al., 2020), (Liu et al., 2019). The first report of human infection was reported from Japan in 2003 (Sugita et al., 2003). Since then, several species, including *M. antarcticus*, *M. parantarctica*, *P. thailandica*, *M. aphidis*, *M. bullatus*, *P. crassa*, and *P. siamensis*, were reported for human infections (Liu et al., 2019). Up to now, a total of 31 cases of patients with Ustilaginales invasion were reported indicating the infectious potential of these plant-pathogenic fungal species (**Table 1**). The species *D. churashimaensis* (previously *Pseudozyma churashimaensis*) was first isolated from leaves of sugarcane in Okinawa, Japan, and described as a new species in 2011 with its host ranging from rice, corn, and sugarcane (Morita et al., 2011). In 2015, by multiloci phylogeny analysis, Wang et al. proposed this fungus to be a new genus *Dirkmeia gen. nov* which only having this species up to now (Wang et al., 2015). In 2020, Anuradha et al. reported 12 cases due to an outbreak of *D. churashimaensis* fungemia in a Neonatal Intensive Care Unit, India, which revealed its pathogenicity in immune suppressed population (Chowdhary et al., 2020). Here, we describe a case of rare subcutaneous infection caused by *D. churashimaensis*. Further, we successfully cured the infection by oral itraconazole treatment.

**Table 1:** Clinical information of Ustilaginales infections in human.

Author, year	Species/Identification	Region	Infection Type/Source	Age/Gender*	Underlying Disease^&^	Clinical Presentation	Treatment^#^	Prognosis
Sugita et al., 2003	*M. antarcticus* *P. thailandica* *M. aphidis*	Northern Thailand	Blood/N/A	NA/M 52/F 21/F	Spontaneous pneumothorax Asthmatic and respiratory failure leptospirosis and aseptic meningitis	N/A	N/A	N/A
Lin et al., 2008	*M. aphidis*	NC USA	Blood/CVC	7/F	Short gut syndrome	Fever, chill, malaise and fatigue	FLC failed→CVC Removal+ oral ITC	Survived
Hwang et al., 2010	*Pseudozyma spp*	Seoul South Korea	Brain Abscess/surgery	78/M	Astrocytoma, MRSA infection	Fever	No AFT	Died
Chen et al., 2011	*M. aphidis*	Wenzhou China	Leg/secondary	51/M	Mycetoma and nocardiosis of leg,	Swollen, discharging sinuses	Oral ITC	Survived
de Carvalho Parahym et al., 2013	*M. aphidis*	Recife Brazil	Pleural fluid/inhalation	17/M	Burkitt lymphoma, chemotherapy	Fever, lung infiltrates	LAMB→VRC	Survived
Prakash et al., 2014	*M. aphidis*	New Delhi India	Blood/N/A	0/M	Hemolytic jaundice	Lethargy, fever, chill	AMB→VRC	Survived
Orecchini et al., 2015	*M. aphidis*	Argentina	Blood/CVC	6/F	Osteosarcoma with lung metastasis, chemotherapy	Fever	LAMB, CVC Removal	Survived
Mekha, 2014	*P. alboarmeniaca* *P. crassa* *P. siamensis*	Nonthaburi Thailand	Blood/N/A	N/A	N/A	N/A	N/A	N/A
Siddiqui, 2014	*Pseudozyma spp*	Georgia USA	Blood/CVC	52/F	Crohn’s disease, colectomy	Fever, headache and weakness	FLC failed→VRC	Survived
Okolo et al., 2015	*M. bullatus*	Córdoba Negeria	Blood/CVC	0/F	Preterm low birth weight	Hypothermia	FLC	Died
Herb et al., 2015	*M. aphidis*	Strasbourg France	Blood/CVC	68/F	Adenocarcinoma of ampulla of Vater, surgery	Fever, chill	LAMB +CVC Removal	Survived
Joo et al, 2016	*M. aphidis*	South Korea	Blood/CVC	51/M	AML, chemotherapy	Fever, lung infiltrate	LAMB+CVC Removal→VRC	Survived
Pande et al., 2017	*Pseudozyma* spp.	Missouri USA	Blood and skin/CVC	44/M	HSCT recipient	High fever	CVC Removal+AmB→VRC	Survived
Liu et al., 2019	*M. antarcticus*	Chengdu, China	Blood	93/M	hypertension, chronic renal insufficiency, Alzheimer’s disease, cerebral infarction, COPD	Fever	CAS(failed)→AMB	Survived
Chowdhary, 2020	*D.churashimaensis* (12 cases)	Delhi, India	blood	neonate	Preterm, low birthweight	N/A	FLU	7 survived, 5 died
This study	*D.churashimaensis* (this study)	Nanchang China	Skin	80/F	No underlying disease	Erythema plaques and nodules	oral ITC	survived

P, pesudozyma; M. Moesziomyces; D. Dirkmiea; USA, the United States.

*In this column, M means male and F means female.

^&^In this column, MRSA means methicillin-resistant Staphylococcus aureus; AML, acute myeloid leukemia; HSCT, haematopoietic stem cell transplantation; COPD, chronic obstructive pulmonary disease.

^#^In this column, → means changing to a new therapy, + means combined therapy. CVC, central venous catheter-related; N/A, not available.; AFT, antifungal treatment; TMP/SMX, trimethoprim/sulfamethoxazole; FLC, fluconazole; AMB, amphotericin B; LAMB, liposomal amphotericin B; VRC, voriconazole; ITC, itraconazole.

## Materials and Methods

### Case Presentation

An 80-year-old female farmer was firstly admitted to our clinic on March 7, 2017 (Dermatology Hospital of Jiangxi Province and Jiangxi Dermatology Institute, Nanchang, South China). Two years prior to her visit, an egg-sized plaque appeared on the extensor side of her left forearm with slight pain, near the wrist joint. The lesion was given no medical attention and slowly spread to the surrounding region. Papules, plaques, and nodules developed successively, with exudation and ulcers appearing on the lesion surface. There was no severe suppuration and sinus tract. Generally, the patient was in a good condition without fever, cough, or fatigue. History of trauma was not recorded, and she had no accompanying systemic diseases or special drug use. She usually works in a farm and get in contact with crops, such as rice and wheat.

**Figure 1:**
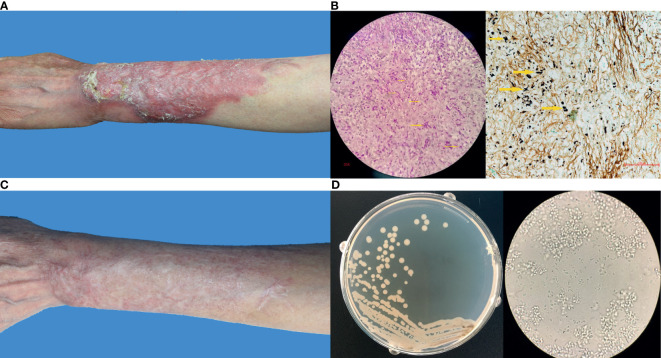
**(A)** Clinical image of this patient before treatment; **(B)** Histopathological examination by Periodic acid–Schiff stain and Methenamine silver stain showing a type of infectious granuloma, abundant blastoconidia, and hyphae elements (yellow arrows indicated); **(C)** Clinical image of this patient after treatment; **(D)** Fungal culture showing yeast-like colonies and budding spores under microscope.

No abnormality was found by biochemical and routine blood examination. CD4+ and CD8+ cell counts were within the normal ranges. The dermatological examination showed a 14 cm × 9 cm irregular infiltrated erythematous plaque on her extensor side of left forearm with distinct margin. Varisized nodes, superficial ulcers, and scales could be observed within the involved region (**Figure 1A**). Moderate to severe pain was reported. Neighboring lymphadenectasis was not discerned.

A skin biopsy was taken from the lesion, and direct examination (KOH 10%) showed fungal spores and conidia. In addition, Periodic acid–Schiff (PAS) and Gomori-Grocott methenamine silver (GM) stains showed a type of infectious granuloma and abundant blastoconidia (**Figure 1B**). Culture of skin biopsy on Sabouraud’s glucose agar (SGA) showed yeast like colonies (**Figure 1D**). Further identification of the fungus was undertaken using ITS sequences using standard primer-pair ITS1 and ITS4. For the identification, a similarity searches with the sequences of ITS regions were done using the BLAST tool against the NCBI database and against the MLST database hosted by Westerdijk Institute, Utrecht, The Netherlands. These BLAST searches revealed that the fungus matched with *Dirkmeia churashimaensis* with 100% similarities (MN515013 and MN515015). The ITS1/2 sequence of the fungus was deposited at GenBank with accession number MK463929.

The *in vitro* antifungal-drug susceptibility test, by the EUCAST. DEF.7.3.1 method, gave a minimum inhibitory concentration (MIC) for fluconazole and 5-flucytocine of 64 mg/L, for ketoconazole and amphotericin B of 0.5 mg/L, for Posaconazole and voriconazole of 2 mg/L, for itraconazole of 1 mg/L, and for terbinafine of 4 mg/L. The case was diagnosed as subcutaneous fungal granuloma by the attending physician, and oral itraconazole (Sporanox) was prescribed with a dosage of 0.2 mg twice a day. Hepatorenal function and whole blood cell analysis were monitored every 2 weeks.

The lesion was healing in the first 2 months of the treatment and dispelled constantly since then. Three months later, all ulcers were cured completely and no new lesions were seen. Consequently, the itraconazole dose was reduced to 0.2 mg per day. The lesion improved continuously and became flattened in the following months. The itraconazole doses was reduced to 0.1 mg each day at 5 months and ended after 6 months of treatment due to the complete relief (**Figure 1C**). The patient tolerated the itraconazole treatment, and no side-effect was reported neither by the patient nor was seen by the physicians. In the 1-year follow-up examination, the patient was free of symptoms.

### Literature Review

For the literature review of clinical reports, we searched in Pubmed, with Google, and in the English language version of the Web of Science database. *Pseudozyma*, *Moesziomyces*, *Dirkmeia*, and *Ustilaginales* were used as keywords, and all reports before April 1, 2021 were included. The etiological agents were undoubtedly identified by morphology and molecular methods. The details of clinical and strain information were retrieved.

## Results

A total of 15 publications including 30 cases were obtained by the search and included, together with the case of this study, in the data analysis. The clinical data were shown in **Table 1**. The information about the strains, including the available antifungal drug resistances data, was summarized in **Table 2**.

**Table 2:** Strain information of Ustilaginales infections in human.

**No.**	**Species/Identification**	**Gene Accession**	**MIC**									**AFST** **Method**
			**FLC**	**ITC**	**VRC**	**AMB**	**CAS**	**5FC**	**Ani**	**Pos**	**Others**	
1	*M. antarcticus*	AB089375(26S) AB089374(ITS)	0.5	0.06	N/A	0.125	N/A	>64	N/A	N/A	N/A	EIKEN kit (Eiken Chemical, Tokyo)
2	*P. thailandica*	AB089355(26S) AB089354(ITS)	2	0.25	N/A	0.25	N/A	>64	N/A	N/A	N/A	EIKEN kit (Eiken Chemical, Tokyo)
3	*M. aphidis*	AB089357(26S) AB089356(ITS)	>64	>8	N/A	0.25	N/A	>64	N/A	N/A	N/A	EIKEN kit (Eiken Chemical, Tokyo)
4	*M. aphidis*	Bankit1010363(ITS)	4	0.125	N/A	0.25	N/A	N/A	N/A	N/A	N/A	YeastOne system (Trek Diagnostic)
5	*Pseudozyma spp*	N/A	N/A	N/A	N/A	N/A	N/A	N/A	N/A	N/A	N/A	N/A
6	*M. aphidis*	N/A	N/A	N/A	N/A	N/A	N/A	N/A	N/A	N/A	N/A	N/A
7	*M. aphidis*	JQ743064(ITS)	4	0.25	0.03	0.25	4	N/A	4	N/A	N/A	CLSI M27-A3
8	*M. aphidis*	KC812275(D1/D2)	8	0.03	0.06	0.03	8	>64	8	0.03	**Isa** 0.25	CLSI M27-A3
9	*M. aphidis*	KM610219(ITS) KM610218(D1/D2)	2	0.03	0.03	0.13	N/A	128	N/A	0.015	N/A	EUCAST E.Def 7.2
10	*P. alboarmeniaca*	AB117961(ITS)	32	4	2	0.25	>16	>64	N/A	N/A	Mica>16	EIKEN kit (Eiken Chemical, Tokyo)
11	*P. crassa*	AB117962(ITS)	>64	>8	2	0.25	>16	>64	N/A	N/A	Mica>16	EIKEN kit (Eiken Chemical, Tokyo)
12	*P. siamensis*	AB117963(ITS)	32	4	2	0.125	>16	>64	N/A	N/A	Mica>16	EIKEN kit (Eiken Chemical, Tokyo)
13	*Pseudozyma spp*	N/A	N/A	N/A	N/A	N/A	N/A	N/A	N/A	N/A	N/A	N/A
14	*M. bullatus*	N/A	128	0.12	0.03	1	8	64	N/A	N/A	N/A	YeastOne Y010
15	*M. aphidis*	N/A	16	0.19	0.03	0.19	>32	>32	N/A	N/A	N/A	EUCAST E.Def 7.2
16	*M. aphidis*	KF443199(ITS) KF443201(D1/D2)	N/A	N/A	N/A	N/A	N/A	N/A	N/A	N/A	N/A	N/A
17	*Pseudozyma* spp.	N/A	N/A	N/A	N/A	N/A	N/A	N/A	N/A	N/A	N/A	N/A
18	*M. antarcticus*	MH185803	128	4	8	<0.5	N/A	>16	N/A	N/A	N/A	ATB FUNGUS 3 (bioMérieux)
19-30	*D. churashimaensis*	N/A	0.157	0.03-0.25	0.03-0.125	0.198	>8	0.157	>8	0.03-0.25	Isa 0.03-0.125	CLSI M27 M60
31	*D. churashimaensis*	MK463929	64	4	2	0.5	N/A	64	N/A	2	KET 0.5	CLSI M27 M60

P, Pesudozyma; M. Moesziomyces; D. Dirkmiea; FLC, fluconazole; ITC, itraconazole; VRC, voriconazole; AMB, amphotericin B; CAS, caspofungin; 5FC, flucytosine; Mica, micafungin; Ani, anidulafungin; Pos, posaconazole; Isa, isavuconazole; KET, ketoconazole; N/A, not available.

The first case of human infection with Ustilaginales species was reported by Sugita et al. in 2003, and three strains were isolated from North Thailand in the year of 2001 which were identified as *M. antarcticus*, *P. thailandica*, and *M. aphidi* (Sugita et al., 2003). We analyzed the 31 cases, including 30 cases from the literature and the case from this study, for the geographic distribution, temporal distribution, species identification, site of infection, clinical manifestation, treatment, and antifungal drug resistance.

Most cases of Ustilaginales infections were reported from Asian countries with 24 cases (77%), 13 from India, 6 from Thailand, 3 from China, 2 from South Korea. There were three cases from the USA and two cases from Africa, one from Argentina, and the other one from Nigeria. Only one case was reported from Europe, from Strasbourg, France. Most of the cases appeared after the year of 2010, taking a percentage of 90% of the reported cases. In the recent 6-year period, 18 cases were reported, which accounts for more than 50% of the reported cases. Fourteen newborns were reported to get infections with Ustilaginales fungi, and the oldest patient was 93 years old (Liu et al., 2019).

Of the 31 isolates, all strains were identified by both phenotype and molecular methods, whereby the sequence of the internal transcribed spacer region (ITS1/2) and D1/D2 region is routinely used for molecular biological species identification. Twenty-eight of the strains were identified to species level, whereas the other 3 were reported as *Pseudozyma* spp. without accession information.

*Dirkmeia churashimanesis* was the predominant clinical species with a percentage of 46% (13/28), and *Moesimyces aphidis* was the second common with 28.6% (8/28) among the identified strains. Additionally, *M. antarctica* two cases were reported and of the other species *M. bullatus*, *P. alboarmeniaca*, *P. crassa*, *P. siamensis*, and *P. thailandica* each one case.

The majority of patients had blood disseminated infections (29/31). Notedly, two of these patients had breaking through infections, with prophylactic treatment of fluconazole and echinocandins (de Carvalho Parahym et al., 2013) (Joo et al., 2016). Two patients in literature had skin infections: one had a secondary to chronic mycetoma and one had a papular eruption over the body with a concurrent blood infection (Chen et al., 2011) (Pande et al., 2017). In this study, we reported the third case of a skin disease caused by *Dirkmeia churashimaensis*. Parahym et al. reported a pulmonary infection of *M. aphidis* from a patient, with a Burkitt lymphoma, during chemotherapy (de Carvalho Parahym et al., 2013). The infection resulted from environmental inhalation. Before the infection occurred, the 17-year-old boy received broad-spectrum antibiotics and antifungal prophylaxis with fluconazole and caspofungin. The blood cultures were negative but the pleural fluid was positive in the fungal examination, and the pathogen was identified as *M. aphidis*. Hwang et al. reported a strain of *Pseudozyma* species isolated from barin abscess (Hwang et al., 2010).

The therapy process for 25 patients was described in the literature as well our study; for six cases, no clinical details were available (Sugita et al., 2003) (Mekha et al., 2014). Eighteen of the 25 patients recovered, and 7 died. Seven patients were prescribed systemic amphotericin B or voriconazole, and all of them recovered finally with good results, among of which three patients were combined with the removal contaminated catheter. Three patients with localized skin and subcutaneous infection completely were cured with oral itraconazole for 2 weeks to 6 months (Chen et al., 2011) (Pande et al., 2017). The therapy of three patients failed with fluconazole (2 cases) or caspofungin (1 case) until changing to the efficient medicines itraconazole, amphotericin B, or voriconazole (Lin et al., 2008) (Siddiqui et al., 2014) (Liu et al., 2019). Furthermore, Chowdhary reported an outbreak of 12 case infections due to *D. churashimanesis* in a neonatal intensive care unit in Delhi India; all patients were treated with fluconazole at a loading dose of 12 mg/kg bodyweight and then 6 mg/kg for 10–14 days; 5 patients died, a case-fatality rate of 42% (Chowdhary et al., 2020). In 2015, Orecchini et al. reported a similar fatal neonate baby as the etiological agent is *M. bullatus* with blood invasion (Orecchini et al., 2015).

Except for these six above deaths, Hwang et al. reported another fatal case. A 78-year-old patient got a brain abscess due to a *Pseudozyma* species in combination with a methicillin-resistant *Staphylococcus aureus* (MRSA) infection after a needle biopsy of brain astrocytoma. The fungal pathogen was identified as *Pseudozyma* strain CBS 10103 (Hwang et al., 2010). The other six dead cases were all preterm, low weight babies who got neonatal sepsis due to *D. churashimanesis* (five cases) (Chowdhary et al., 2020) and *M. bullatus* (one case) (Orecchini et al., 2015). These babies died despite fluconazole treatment. For 26 of the strains, antifungal drug resistances data were available (**Table 2**). Most strains had low MICs to itroconazole, voriconazole, and amphotericin B, whereas high MICs to fluconazole (0.5–128 mg/L), flucytosine (>64 mg/L), caspofungin (4–32 mg/L), and micafungin (>16 mg/L). However, in Chowdhary’s report (Chowdhary et al., 2020), all the 12 *D. churashimanesis* strains showed sensitive to azoles, including itraconazole, fluconazole, voriconazole, Posaconazole, and isavuconazole, with low MICs. Amphotericin B and 5-flucytosine also had potent activity. The resistance to echinocandins, including caspofungin, anidulafungin, and micafungin (MICs > 8 mg/L), is coincident with other strains.

## Discussion

In this paper, we reported a severe subcutaneous infection caused by *D. churashimaensis* and performed a literature review of human disease caused by Ustilaginales fungi. By analyzing the demographic, clinical, and strain information, we got a better understanding of their geographical distribution, susceptible population, and susceptibility to routine antifungal drugs. Our patient presented an extensive granuloma on her forearm, and the pathogenic agent was identified as *D. churashimaensis* by morphology and molecular methods. After long-term treatment with itraconazole for 6 months with a total dose of 49.5 grams, the patient recovered completely.

The species *D. churashimaensis* (previously *Pseudozyma churashimaensis*) was first described in 2011 from the leaves of sugarcane in Japan. By multigene phylogeny analysis combined ITS and LSU rRNA gene, Wang et al. proposed it to be a new genus, *Dirkmeia* which belongs to an isolated branch in the Ustilaginales (Wang et al., 2015). In our case, the yeast form of this fungus was highly virulent and invaded the epidermis and dermis layers, which lead to a very extensive damage within 2 years. However, our strains presented as cream-colored yeast colony without blackish to brownish powdery spores whose synthesis perhaps was blocked by unappropriated environment in skin tissue. Chowdhary et al. reported an outbreak of 12 cases due to this fungus in NICU, in India last year (Chowdhary et al., 2020). All the patients were preterm neonates with other risk factors, including central venous catheter, persistent hypoglycemia, severe asphyxia, sepsis, and mechanical ventilation. All samples were isolated from blood and grew as yeast-like cream to pale yellow. In our case, the patient had no obvious immunocompromised status. Considering she usually works in gardens and farms, we speculate that chronic damage of the skin was due to farm work in combination with chronic exposure to the opportunist which cause the infection. Our study together with Chowdhary et al. reported the significance of the *D. churashimaensis* as opportunistic fungi in human hosts.

We also reviewed 31 clinical cases caused by the following fungi including *D. churashimaensis* and *M. aphidis* (synonym to *P. aphidis*) which were the most common agents, responsible for 75% of all strains identified to species level. In addition, some other species were also reported including *M. antarcticus*, *P. thailandica*, *M. parantarctica*, *P. alboarmeniaca*, *P. crassa*, *P. siamensis*, and *M. bullatus*.

Smut fungi are usually found in the environment and can be transferred to human by direct and indirect contact. Catheter-related infections were common invasive route and could lead to blood dissemination for invasive yeast infections (Pappas et al., 2015). Removals of the contaminated catheter were strongly suggested for catheter-related infections of candidemia (Pappas et al., 2015). In our review, catheter removal was explored to three cases with proper antifungal therapy and the infection vanished. In three of these patients, locally skin irritations and in one patient cutaneous rash were diagnosed and were related to the blood infection. Infections by inhalation were not common and, in this review, only one case got pulmonary invasion during chemotherapy (de Carvalho Parahym et al., 2013). Furthermore, one brain abscess occurred after traumatic examination (Hwang et al., 2010).

Generally, underlying immune deficiency was high-risk factor for invasive fungal infections. Among those with detailed clinical information, 29 of 31 patients had a local barrier damage (including pneumothorax, surgery, and basic dermatosis) or systemic immunodeficiency including preterm, low birth weight, Crohn’s disease, short gut syndrome, malignant cancer, and chemotherapy. With the rise of the immunocompromised population, the overuse of antibiotics and increase of invasive operations, clinical cases due to unusual saprophytic fungi have increased during the last few years (Skiada et al., 2017). Especially in immunocompromised patient, saprophytic fungal species take the opportunity to invade the host and as the boundary between saprophytic and pathogenic are less clear in these cases. Diagnosis of unusual fungal infections should be done regularly, and drug susceptibility test should be performed for all of this these strains.

For these strains within the genus *Dirkmeia*, *Pseudozyma*, and *Moesziomyces*, amphotericin B and azoles (ketoconazole, posaconazole, itraconazole and voriconazole) have a good antifungal activity. Doing antifungal susceptibility testing might help in choosing proper therapy for these kinds of infections. All *Dirkmeia* isolates from Chowdhary’s study showed potent activity of fluconazole (MIC 1–4 μg/ml; GM 2.37 μg/ml) and 5-flucytosine (GM MIC: 0.157 μg/ml). Although, in the other studies, the MICs of fluconazole and 5-flucytosine show high MICs, the former with a range of 2–128 mg/L and the latter higher than 32 mg/L (Chowdhary et al., 2020). Considering the high percentage of death especially from those who received fluconazole therapy and there were two breakthrough infections occurred with prophylactic use of fluconazole as fluconazole seems not efficient for these kinds of infections. Therefore, *in vitro* antifungal test with more clinical strains of Ustiginales is needed in future. Caspofungin, micafungin, and anidufungin had high MICs for all species and always failed as therapeutic against Ustilaginales infection. Considering that echinocandins are commonly used for treating of invasive *Candida* infection, the attending physician should be aware of the possibility of a Ustilaginales infections.

Itraconazole is a broad-spectrum antifungal agent commonly used for subcutaneous fungal infection and applied with an empirical dose of 0.4 mg/day. In our case, the *in vitro* and *in vivo* efficiency of itraconazole correlations and our results is in agreement with previous published guidelines of subcutaneous fungal infections (Kauffman et al., 2007). Most of cases from the literature especially with local skin infection were successfully treated with oral itraconazole. A high dose at the beginning of the therapy increases quickly the concentration in the blood and inhibits the fungal growth. When the lesion is under controlled, the dosage can be adjusted according to the clinical prognosis and adverse reactions. Our patient treated with itraconazole lasted for 6 months, and no relapse was seen in the 1 year follow up control examination.

In conclusion, we report a case of human infection due to *D. churashimaensis*. The patient, an 80-year-old woman, developed plaques and nodules on her left arm. Oral treatment with itraconazole was successful against the infection. An anti-fungal drug susceptibility test of *D. churashimaensis* and a literature review indicates that itraconazole could be the first choice for the therapy against skin/subcutaneous *D. churashimaensis* infections. Since some of the common anti-fungal drugs, for example; fluconazole and echinocandins, are ineffective against *Dirkmeia* species, we here highlight the importance of proper identification of the causative agents. Furthermore, antifungal susceptibility tests should be done regularly.

## Data Availability Statement

The datasets presented in this study can be found in online repositories. The names of the repository/repositories and accession number(s) can be found in the article/supplementary material.

## Ethics Statement

Written informed consent was obtained from the individual(s) for the publication of any potentially identifiable images or data included in this article.

## Author Contributions

FH and PW contributed to diagnosing, deciding treatment, and follow-up. LZ contributed to the pathological examinations. QJ contributed to the fungal identification. CW, AA-H, OB, and PZ contributed to manuscript writing and revisions and approved the final manuscript. All authors contributed to the article and approved the submitted version.

## Funding

This work was supported by National Natural Science Foundation of China (81960367) and the Natural Science Foundation of Shandong Province of China (ZR2017MH121).

## Conflict of Interest

The authors declare that the research was conducted in the absence of any commercial or financial relationships that could be construed as a potential conflict of interest.

## Publisher’s Note

All claims expressed in this article are solely those of the authors and do not necessarily represent those of their affiliated organizations, or those of the publisher, the editors and the reviewers. Any product that may be evaluated in this article, or claim that may be made by its manufacturer, is not guaranteed or endorsed by the publisher.

## Abbreviations

SGA, Sabouraud’s glucose agar; CD4, cluster of differentiation 4; CD8, cluster of differentiation 8; MIC, Minimum inhibitory concentration; KOH, Potassium Hydroxide; PAS, Periodic acid–Schiff; GM, Gomori-Grocott methenamine.

## References

[B1] Chen B. Zhu L. Y. Xuan X. Wu L. J. Zhou T. L. Zhang X. Q.. (2011). Isolation of Both Pseudozyma Aphidis and Nocardia Otitidiscaviarum From a Mycetoma on the Leg. Int. J. Dermatol. 50, 714–719. doi: 10.1111/j.1365-4632.2010.04814.x 21595667

[B2] Chowdhary A. Sharada K. Singh P. K. Bhagwani D. K. Kumar N. de Groot T.. (2020). Outbreak of Dirkmeia Churashimaensis Fungemia in a Neonatal Intensive Care Unit, India. Emerg. Infect. Dis. 26, 764–768. doi: 10.3201/eid2604.190847 32186501 PMC7101094

[B3] de Carvalho Parahym A. M. R. da Silva C. M. de Farias Domingos I. Gonçalves S. S. de Melo Rodrigues M. de Morais V. L. L.. (2013). Pulmonary Infection Due to Pseudozyma Aphidis in a Patient With Burkitt Lymphoma: First Case Report. Diagn Microbiol. Infect. Dis. 75, 104–106. doi: 10.1016/j.diagmicrobio.2012.09.010 23182077

[B4] Herb A. Sabou M. Delhorme J. B. Pessaux P. Mutter D. Candolfi E.. (2015). Pseudozyma Aphidis Fungemia After Abdominal Surgery: First Adult Case. Med. Mycol. Case Rep. 8, 37–39. doi: 10.1016/j.mmcr.2015.03.001 25870786 PMC4389203

[B5] Hwang S. Kim J. Yoon S. Cha Y. Kim M. Yong D.. (2010). First Report of Brain Abscess Associated With Pseudozyma Species in a Patient With Astrocytoma. Korean J. Lab. Med. 30, 284–288. doi: 10.3343/kjlm.2010.30.3.284 20603589

[B6] Joo H. Choi Y. G. Cho S. Y. Choi J. K. Lee D. G. Kim H. J.. (2016). Pseudozyma Aphidis Fungaemia With Invasive Fungal Pneumonia in a Patient With Acute Myeloid Leukaemia: Case Report and Literature Review. Mycoses 59, 56–61. doi: 10.1111/myc.12435 26608844 PMC4738435

[B7] Kauffman C. A. Bustamante B. Chapman S. W. Pappas P. G. (2007). Clinical Practice Guidelines for the Management of Sporotrichosis: 2007 Update by the Infectious Diseases Society of America. Clin. Infect. Dis. 45, 1255–1265. doi: 10.1086/522765 17968818

[B8] Kruse J. Doehlemann G. Kemen E. Thines M. (2017). Asexual and Sexual Morphs of Moesziomyces Revisited. IMA Fungus 8, 117–129. doi: 10.5598/imafungus.2017.08.01.09 28824844 PMC5493530

[B9] Lin S. S. Pranikoff T. Smith S. F. Brandt M. E. Gilbert K. Palavecino E. L.. (2008). Central Venous Catheter Infection Associated With Pseudozyma Aphidis in a Child With Short Gut Syndrome. J. Med. Microbiol. 57, 516–518. doi: 10.1099/jmm.0.47563-0 18349374

[B10] Liu Y. Zou Z. Hu Z. Wang W. Xiong J. (2019). Morphology and Molecular Analysis of Moesziomyces Antarcticus Isolated From the Blood Samples of a Chinese Patient. Front. Microbiol. 10, 254. doi: 10.3389/fmicb.2019.00254 30828326 PMC6384246

[B11] Mekha N. Takashima M. Boon-long J. Cho O. Sugita T. (2014). Three New Basidiomycetous Yeasts, Pseudozyma Alboarmeniaca Sp. Nov., Pseudozyma Crassa Sp. Nov. And Pseudozyma Siamensis Sp. Nov. Isolated From Thai Patients. Microbiol. Immunol. 58, 9–14. doi: 10.1111/1348-0421.12111 24215507

[B12] Morita T. Ogura Y. Takashima M. Hirose N. Fukuoka T. Imura T.. (2011). Isolation of Pseudozyma Churashimaensis Sp. Nov., a Novel Ustilaginomycetous Yeast Species as a Producer of Glycolipid Biosurfactants, Mannosylerythritol Lipids. J. Biosci. Bioeng 112, 137–144. doi: 10.1016/j.jbiosc.2011.04.008 21606002

[B13] Ojogba M. O. Anne D. Bose T. Nnaemeka E. N. Mebi G. A. Ikenna K. O.. (2015). First Report of Neonatal Sepsis due to Moesziomyces bullatus in a Preterm Low-Birth-Weight Infant. JMM Case Rep. 2015, 1–4. doi: 10.1099/jmmcr.0.000011

[B14] Okolo O. M. Van Diepeningen A. D. Toma B. Nnadi N. E. Ayanbimpe M. G. Onyedibe I. K.. (2015). First Report of Neonatal Sepsis Due to Moesziomyces bullatus in a Preterm Low-Birth-Weight Infant. JMM Case Reports. 2 (2). doi: 10.1099/jmmcr.0.000011

[B15] Orecchini L. A. Olmos E. Taverna C. G. Murisengo O. A. Szuzs W. Vivot W.. (2015). First Case of Fungemia Due to Pseudozyma Aphidis in a Pediatric Patient With Osteosarcoma in Latin America. J. Clin. Microbiol. 53, 3691–3694. doi: 10.1128/JCM.01095-15 26292313 PMC4609714

[B16] Pande A. Non L. R. Romee R. Santos C. A. Q. (2017). Pseudozyma and Other non- Candida Opportunistic Yeast Bloodstream Infections in a Large Stem Cell Transplant Center. Transpl Infect. Dis. 19, e12664. doi: 10.1111/tid.12664 28099778

[B17] Pappas P. G. Kauffman C. A. Andes D. R. Clancy C. J. Marr K. A. Ostrosky-Zeichner L.. (2015). Clinical Practice Guideline for the Management of Candidiasis: 2016 Update by the Infectious Diseases Society of America. Clin. Infect. Dis. 62, e1–e50. doi: 10.1093/cid/civ933 26679628 PMC4725385

[B18] Prakash A. Wankhede S. Singh P. K. Agarwal K. Kathuria S. Sengupta S.. (2014). First Neonatal Case of Fungaemia Due to Pseudozyma Aphidis and a Global Literature Review. Mycoses 57, 64–68. doi: 10.1111/myc.12098 23834440

[B19] Siddiqui W. Ahmed Y. Albrecht H. Weissman S. (2014). Pseudozyma Spp Catheter-Associated Blood Stream Infection, an Emerging Pathogen and Brief Literature Review. BMJ Case Rep. 2014, 10–12. doi: 10.1136/bcr-2014-206369 PMC4267066 25498807

[B20] Skiada A. Pavleas I. Drogari-Apiranthitou M. (2017). Rare Fungal Infectious Agents: A Lurking Enemy. F1000Research 6, 1–17. doi: 10.12688/f1000research.11124.1 PMC5664977 29152230

[B21] Sugita T. Takashima M. Poonwan N. Mekha N. Malaithao K. Thungmuthasawat B.. (2003). The First Isolation of Ustilaginomycetous Anamorphic Yeasts, Pseudozyma Species, From Patients’ Blood and a Description of Two New Species: P. Parantarctica and P. Thailandica. Microbiol. Immunol. 47, 183–190. doi: 10.1111/j.1348-0421.2003.tb03385.x 12725287

[B22] Telles J. P. Ribeiro V. S. T. Kraft L. Tuon F. F. (2020). Pseudozyma Spp. Human Infections: A Systematic Review. Med. Mycol 59, 1–6. doi: 10.1093/mmy/myaa025 32343341

[B23] Wang Q. M. Begerow D. Groenewald M. Liu X. Z. Theelen B. Bai F. Y.. (2015). Multigene Phylogeny and Taxonomic Revision of Yeasts and Related Fungi in the Ustilaginomycotina. Stud. Mycol. 81, 55–83. doi: 10.1016/j.simyco.2015.10.004 26955198 PMC4777779

